# Quantity and quality of image artifacts in optical coherence tomography angiography

**DOI:** 10.1371/journal.pone.0210505

**Published:** 2019-01-25

**Authors:** Christian Enders, Gabriele E. Lang, Jens Dreyhaupt, Max Loidl, Gerhard K. Lang, Jens U. Werner

**Affiliations:** 1 Department of Ophthalmology, Ulm University, Ulm, Germany; 2 Institute of Epidemiology and Medical Biometry, Ulm University, Ulm, Germany; Massachusetts Eye & Ear Infirmary, Harvard Medical School, UNITED STATES

## Abstract

**Objective:**

To analyze quality and frequency of OCTA artifacts and to evaluate their impact on the interpretability of OCTA images.

**Design:**

75 patients with diabetic retinopathy (DR), retinal artery occlusion (RAO), retinal vein occlusion (RVO), or neovascular age-related macular degeneration (nAMD) and healthy controls were enrolled in this cross-sectional study in the outpatient department of a tertiary eye care center.

**Methods:**

All participants underwent an OCTA examination (spectral domain OCT Cirrus 5000 equipped with the AngioPlex module). OCTA scans were analyzed independently by two experienced ophthalmologists. Frequency of various artifacts for the entire OCTA scan and for different segmentation layers and the grading of OCTA interpretability were investigated.

**Results:**

The analysis of 75 eyes of 38 women and 37 men between 24 and 94 years were included. Six eyes had no retinal disease, 19 eyes had nAMD, 16 had DR, 19 eyes had RVO, and 15 eyes showed RAO. A macular edema (ME) was present in 40 of the diseased eyes. Projection artifacts occurred in all eyes in any structure below the superficial retinal vessel layer, segmentation and motion artifacts were found in 55% (41/75) and 49% (37/75) of eyes, respectively. Other artifacts occurred less frequently. Segmentation artifacts were significantly more frequent in diseased than in healthy eyes (p<0.01). Qualitative assessment of OCTA images was graded as excellent in 65% and sufficient in 25% of cases, adding up to 91% images deemed acceptable for examination. Presence of ME was associated with a significantly poorer interpretability (p<0.01).

**Conclusion and Relevance:**

Various artifacts appear at different frequencies in OCTA images. Nevertheless, a qualitative assessment of the OCTA images is almost always possible. Good knowledge of possible artifacts and critical analysis of the complete OCTA dataset are essential for correct clinical interpretation and determining a precise clinical diagnosis.

## Introduction

Optical coherence tomography angiography (OCTA) represents a functional extension of the structural, intensity-based OCT and enables a non-invasive, quick, microvascular 3D-visualization of the retina and choroid in without the use of a contrast agent. In principle, it detects the movement of scattering particles (e.g. red-blood cells) within sequential optical coherence tomography (OCT) B-scans performed repeatedly at the same location of the retina. Changes in temporal contrast at a specific location indicate movement (erythrocyte motion) and hence vessel location. It has already been demonstrated that OCTA facilitates the exact diagnosis and follow-up of vascular diseases in the posterior segment of the eye, as essential findings on both morphology and perfusion status are obtained simultaneously. Accordingly, this technology has great potential in the diagnosis and follow-up of various retinal and choroidal diseases such as diabetic retinopathy (DR), retinal vascular occlusion and age-related macular degeneration (AMD) [1–3].

However, as with any other imaging method, artifacts also occur in OCTA [4,5]. Artifacts can lead to misdiagnosis and unnecessary treatment [6]. Three main groups of artifacts can be distinguished: (a) artifacts that are inherent in the OCTA technology independently of the device type used, (b) artifacts due to algorithms for data acquisition and image processing and (c) motion-related artifacts [6,7].

As artifacts can affect OCTA interpretation, the aim of our study was to analyze the quantity and severity of OCTA artifacts in a routine clinical setting and to evaluate the effect of their impairment on the diagnostic value of OCTA images.

## Design and methods

We designed a prospective cross-sectional study with 75 consecutive patients and healthy controls who underwent an OCTA examination between October 2016 and January 2017 at the outpatient department of an ophthalmological tertiary care center at an academic hospital. Approval for our study was granted by the local Ethics Committee (approval by Institutional review board of the University, application number 388/15). Patient informed consent was obtained prior to their enrolment.

### Participants

Seventy-five patients with either DR, retinal artery occlusion (RAO), retinal vein occlusion (RVO), or neovascular AMD (nAMD) and healthy controls were included in the study, if they were older than 18 years, phakic or pseudophakic. Subsequently, an 8 mm x 8 mm OCTA scan centered on the fovea was performed. The retinal disease had been diagnosed prior to the OCTA examination during a routine consultation. Exclusion criteria were retinal diseases other than DR, RAO, RVO or nAMD, loss of visual fixation, or a significant degree of opacification. Healthy controls had no retinal disease in either eye and had solely been referred to our center for an ophthalmologic pathology in the anterior segment of the contralateral eye. OCTA recordings with a signal strength below 6 were excluded to ensure sufficient image quality.

### Assessments

All patients received a complete ophthalmologic examination including thorough history-taking, best corrected visual acuity (BCVA) and funduscopy by an ophthalmologist. OCT and OCTA examination were performed by a very experienced and well-trained technician. When necessary, also fundus photography or fluorescein angiography was performed. OCTA examinations were carried out with the spectral domain OCT CIRRUS HD-OCT model 5000 equipped with the AngioPlex module, Software version 9.5.1.13585 (Carl Zeiss Meditec, Inc., Dublin, USA). The CIRRUS 5000 utilizes a line-scanning ophthalmoscope (LSO) and operates at scanning speeds up to 68 KHz. The FastTrac eye tracking system reliably tracks even small eye movements. All in all, this provides a high-contrast image of the fundus and a good superimposition of different scans of a subject’s eye, which contributes to a reduction of movement artifacts. The AngioPlex software is based on "Optical Micro Angiography Complex”(OMAGc) algorithms, that leverages both amplitude and phase OCT full spectrum signal data to precisely evaluate contrast differences between consecutive B-scans. This is the basis to provide three dimensional images of microvasculature of retina and choroid in high-resolution [8]. Automatically, the AngioPlex module extracts different slabs of interest from the data cube. Each slab represents the collection of B-scans across a specific depth in the retina, thus providing a visualization of the retinal vasculature in this segmentation. En face slabs of the following segmentations are provided automatically: superficial retinal layer, deep retinal layer, avascular layer, choriocapillary layer and choroidal layer. This technology has been described in detail previously [1,8].

**Fig 1:**
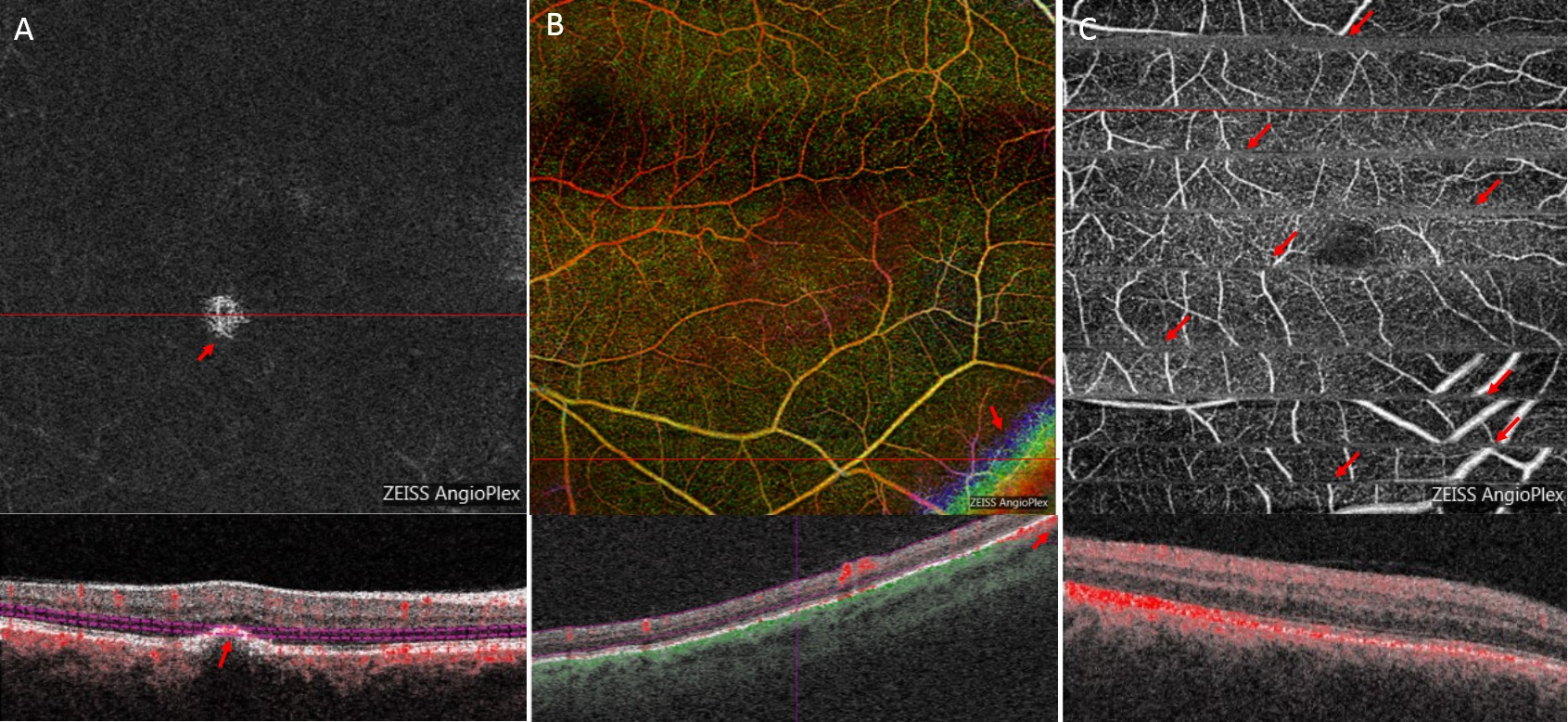
Examples of artifacts. For each artifact OCTA enface and B-Scans are shown. Red arrows indicate the artifacts, red lines indicate the corresponding B-scan. (A) segmentation artifact retina avascular layer. (B) out of window artifact retina layer depth encoded. (C) motion artifact retina layer.

In order to detect artifacts, 8x8 mm volume scans around the fovea were independently analyzed by two experienced, board-certified ophthalmologists (CE, JUW). For the analysis, the entire OCT/OCTA data set was carefully evaluated, also taking into account results obtained from ophthalmoscopic findings, fundus photography and fluorescence angiography. Moreover, presence of relevant macular edema (ME) was documented. Central subfield foveal thickness (CSFT) greater or less than 400 micrometers was assessed in cases positive for ME. Artifacts were defined according to consensus in the published literature [1,4,5]. All OCTA scans were checked for artifacts in all segmentations such as segmentation artifacts, projection, motion, banding, blink, masking, out-of-window, vessel doubling, and stretch artifacts. A tabular overview of artifacts including their cause and definition is shown in Table 1, examples of artifacts are shown in Fig 1 and S1 Fig. Finally the two independent investigators graded the general interpretability of the OCTA images on an ordinal scale („excellent“, „sufficient”and „poor“). If the results differed between the two investigators, a third independent investigator was consulted to act as an umpire (GEL).

**Table 1:** Overview of artifacts including their cause and definition.

Artifact	Cause and Definition
**projection artifact**	Vascular structures of superficial layers are also displayed incorrectly in deeper layers.
**masking**	Dense media may lead to signal loss in underlying layers and impede their visualization.
**segmentation artifact**	Errors in (automatic) segmentation may lead to incorrect OCTA results.
**out of window artifact**	Loss of scan focus in certain retinal areas, e.g. due to tumour, high myopia. Easily recognizable in the OCT image.
**Vessel doubling**	As a result of image processing, vessels are displayed twice directly next to each other within a layer.
**motion artifact**	As a result of eye movements, very thin white horizontal lines resulting in an illusive interruption or displacement of the vessels.
**blink artifact**	Vertical and horizontal black lines in each layer, caused by blinking.
**banding artifact**	Adjacent horizontal stripes of different brightness.
**stretch artifact**	Short stripes of different brightness at the edges of OCTA images.

### Statistical methods

The study design is explorative. The sample size was chosen according to considerations of feasibility. A sample of 75 patients was considered a sufficient sample size regarding the planned analysis. All patients were recruited between October 2016 and January 2017.

For analysis we used statistical methods for categorical data to test associations. Therefore, associations between categorical variables (diagnosis, type of artifact, signal strength of OCTA recordings (6–8 vs 9–10), assessability of OCTA images (poor, sufficient, excellent), and presence of ME (none, CSFT < 400 μm, CSFT > 400 μm) were assessed using the χ^2^-test or Fisher’s exact test as appropriate. Because of the explorative nature of this study, no adjustment for multiple testing was made. A two-sided P value <0.05 was considered significant. The results of all statistical tests were interpreted in an exploratory manner. Statistical analyses were conducted using SAS, version 9.4 (SAS Institute Inc, Cary NC).

## Results

Seventy-five eyes of 38 women and 37 men with a mean age of 66 ± 16 years (range 24–94) were studied. Of these, 6 ophtalmological assessments did not show any retinal disease and were included as controls, 19 showed nAMD, 16 had DR, 19 had RVO, and 15 had RAO. A macular edema was present in 40 of our cases. Table 2 shows the frequency and severity of ME in the found for each diagnosis.

**Table 2:** Absolute frequency and central thickness of macular edema by diagnosis.

	ME CSFT > 400 μm	ME CSFT < 400μm	no ME
**DR (n = 16)**	6	3	7
**nAMD (n = 19)**	4	5	10
**RVO (n = 19)**	15	2	2
**RAO (n = 15)**	2	3	10
**Control (n = 6)**	0	0	6
**Total (n = 75)**	27	13	35

ME: macular edema;CSFT: central subfield foveal thickness;DR: diabetic retinapathy; nAMD: neovascular age-related macular degeneration; RVO: retinal vein occlusion; RAO: retinal artery occlusion

**Fig 2:**
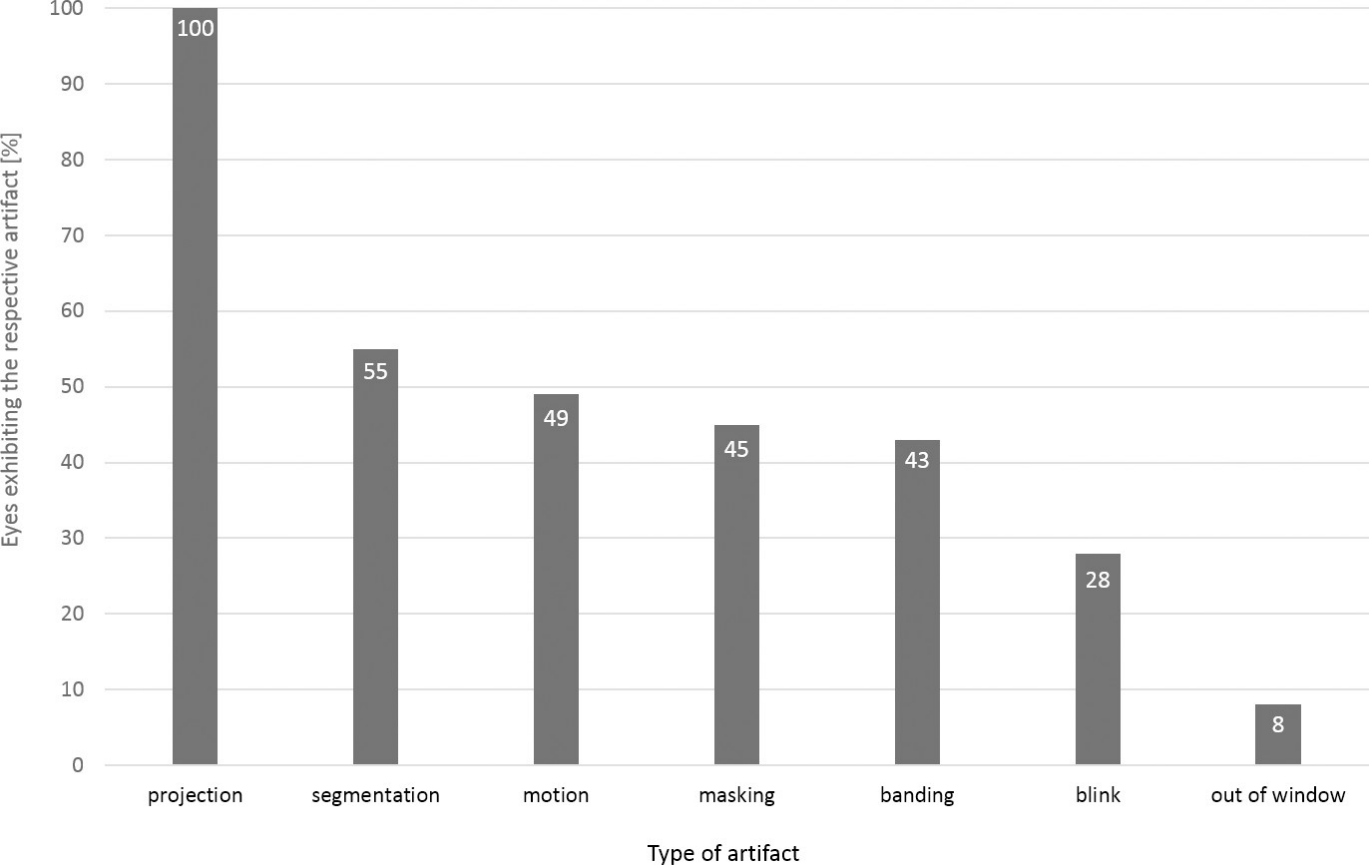
Different types of identified artifacts and their relative frequencies per entire OCTA scan.

**Fig 3:**
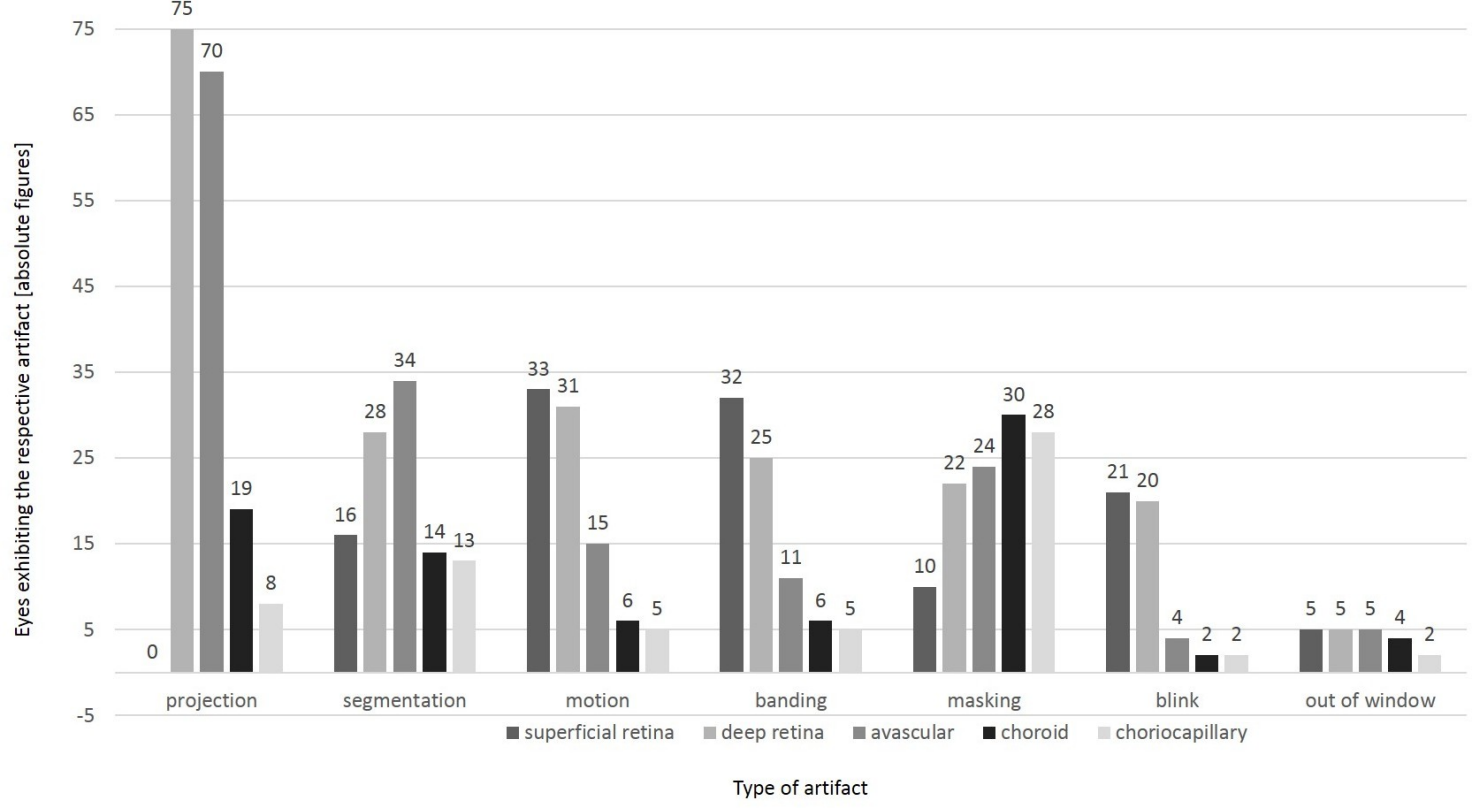
Frequencies of various artifacts in different segmentations of the OCTA scans. Overall, the interpretability of the OCTA images was graded sufficient in 25% of assessments (19/75, 95% confidence interval (CI): 16%-37%) and excellent in 65% (49/75, 95% CI: 53%-76%), adding up to 91% of OCTA assessments deemed of an acceptable quality allowing for clinical interpretation (68/75, 95% CI: 82%-96%). Also, the presence of macular edema was associated with poorer interpretability: While 85.7% of OCTA images of eyes without ME were excellently interpretable, 76.9% of OCTA imaging results with a ME < 400 μm and 33.3% with a ME > 400 μm (p<0.01; Fisher`s exact test) were deemed of sufficient quality for clinical interpretability.

Artifact frequencies varied. While projection artifacts were observed in 75/75 OCTA assessments, segmentation artifacts occurred in 41/75 cases, followed by motion artifacts in 37/75 cases. The least frequent artifacts were out of window artifacts in 7/75 cases. Vessel doubling and stretch artifacts were not detected. Novel artifacts were also not identified. Types of artefacts per case are displayed in Fig 2. Furthermore, the frequency distribution of different artifact types varied per segmentation layer. Motion, banding, and blink artifacts occurred more frequently in the upper retinal layers such as the superficial retinal layer and the deep retinal layer, while segmentation and projection artifacts were mainly found in the deep retinal layer and the avascular retinal layer. Masking artifacts predominantly occurred in the choroid layer. Fig 3 shows the frequencies of various artifacts in different segmentations. While segmentation artifacts (p< 0.01; χ^2^-test) were significantly more common in OCTA examination of eyes with underlying pathologies, no significant difference could be detected for the distribution of all other artifact types between OCTA assessments of cases with underlying ophthalmological pathologies or healthy controls.

## Discussion

As in any other imaging technology of the retina and choroid, artifacts are quite common in OCTA images as well. However, even though artifacts occurred frequently in our study, this did not affect the general interpretability of the OCTA images: In 91% of the eyes examined, the OCTA dataset allowed for a sufficient to excellent interpretation of the retinal microvasculature and provided important insights into morphology and perfusion status. Artifacts in OCTA may be caused due to the method of data acquisition, data processing algorithms, certain properties of the eye, pathological alterations, and insufficient cooperation of the patient during image capture [4,5,7]. In our study, the various types of artifacts were differently distributed over the various layers of OCTA images (Fig 3). Moreover, they differed in terms of their identifiability as artifacts. Motion artifacts and blink artifacts predominantly occur within the upper layers (superficial and deep retina layers; Fig 3), and are in general clearly identifiable as artifacts as such.

In contrast, segmentation, projection and masking artifacts can lead to abnormal structures that seem to occur in layers where they are not actually present. Therefore, particular attention should be paid to these artifacts when analyzing OCTA data to ensure correct clinical evaluation.

Of these, projection artifacts occurred most frequently and were present in all subjects in all structures below retinal vessels in our study. They should therefore generally be considered when interpreting OCTA data, especially in areas with vessels above them [4]. Therefore, an analysis of the entire OCTA data set should be used to critically examine whether identical, characteristic vascular patterns occur repeatedly in several layers of the retina. Currently, manufacturers of OCTA devices are working on more efficient algorithms to reduce projection artifacts [9]. Alternatively to the conventional projection removal by slab subtraction or masking, which was adopted by AngioVue (Optovue, Fremont, CA) and AngioPlex (Zeiss Medical Technology, Dublin, CA) commercial systems, Patel proposed a projection-resolved OCTA (PR-OCTA) algorithm that resolves the ambiguity between in situ flow and projection artifact at the level of single volumetric pixels [10].

Moreover, our results as well as those of other publications suggest that segmentation artifacts occur more frequently in eyes with pathologies than in healthy eyes [11]. Even if general interpretability was not limited, segmentation artifacts occurred relatively frequently (41/75; 55%) in our study, mainly in the avascular retina and deep retinal layer. This might be due to the fact, that in case of severe pathological changes the structures required as a reference value for automatic segmentation are no longer reliably recognized by the software. The selected segmentation lines should therefore be checked for plausibility in the corresponding B-scan and, if necessary, adjusted to ensure correct interpretation. Accordingly, especially in cases of pronounced pathological changes of the retina and choroid, an operator interaction is of utmost importance to check the segmentation lines for plausibility. This contributes to reduction of segmentation artifacts and may help to ensure correct evaluation and diagnosis.

Also masking artifacts were significantly more frequent in OCTA assessments of pathologically altered eyes in our study and occurred in all layers of the scan with roughly the same frequency. This is also conclusive, since vitreous floaters or vitreous hemorrhages can cause masking artifacts in the upper retinal layers, while subretinal bleeding can affect the visualization of vessels in the choriocapillaris and choroid. Also pronounced edema and even highly reflective layers such as RPE or fibroses can make the visualization of the underlying layers more difficult due to masking effects. Therefore, OCTA datasets should always be analyzed in adjunction with fundus images in order to exclude artifacts caused by overlaying structures.

Some artifacts that have previously been described in the literature such as unmasking, vessel doubling, stretching, and crisscross artifacts were not identified in our study. This might be due to the chosen patients concerning unmasking or the improved image and data processing algorithms that are now used in the current AngioPlex software.

Even if artifacts occurred frequently in our analysis, this did not affect the general interpretability of the OCTA images which were deemed excellent or sufficient in 91% of cases. As expected, the presence of a ME impeded the evaluation of OCTA images.

Our study has limitations that should be addressed. First, the number of participants was limited due feasibility constraints. Furthermore, this study is explorative in nature. However, we assume that this number is sufficient to achieve meaningful findings. Secondly, we included only patients with one of four retinal diagnoses and healthy patients who were examined consecutively at our tertiary care center. This does not necessarily represent real-life incidence and may limit the generalization of our results. In addition to our present study, we suggest future investigations on the correlation of visual acuity and interpretability of OCTA images to improve the quality of retinal diagnostics.

## Conclusion

As in any other imaging procedure, various artifacts appear in OCTA images with different frequencies. Nevertheless, a qualitative assessment of the OCTA images is almost always possible and can provide the clinician with valuable insights into the morphology and perfusion status of the choroid and retina. In order to maximize the benefit from OCTA images, operator interaction is required to reduce the frequency of artifacts. Moreover, a good knowledge of possible artifacts and a critical analysis of the complete OCTA dataset also in adjunction with fundus photography are essential for correct clinical interpretation and precise clinical diagnosis. New algorithms for data and image processing will possibly contribute to a reduction of artifacts in the future.

## Supporting information

S1 Fig More examples of OCTA artifacts. For each artifact OCTA enface and B-Scans are shown. Red arrows indicate the artifacts, red lines indicate the corresponding B-scan. (A) Projection artifact. (B) Masking artifact. (C) banding artifact. (D) Blink artifact.
(TIF)
