# Supplementary figures and images for: Quantity and quality of image artifacts in optical coherence tomography angiography
Source: PLoS One. 2019 Jan 25;14(1):e0210505. doi: 10.1371/journal.pone.0210505 (PMC6347178; doi:10.1371/journal.pone.0210505)

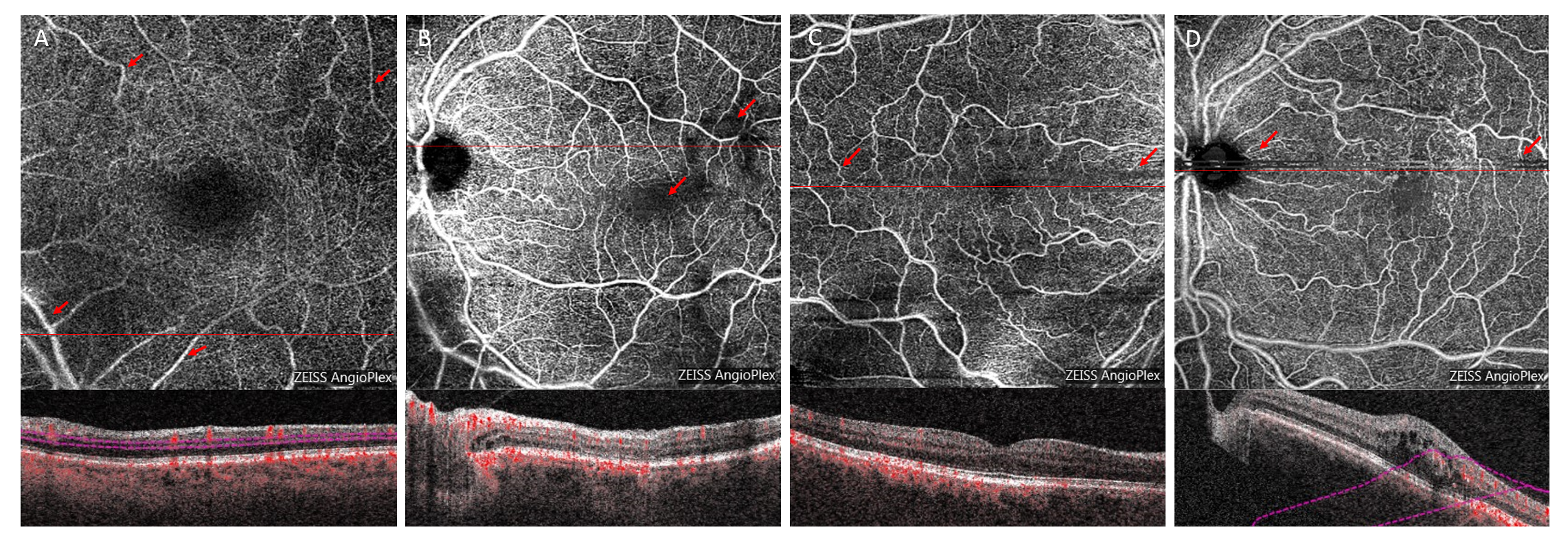

Supplement: S1 Fig — For each artifact OCTA enface and B-Scans are shown. Red arrows indicate the artifacts, red lines indicate the corresponding B-scan. (A) Projection artifact. (B) Masking artifact. (C) banding artifact. (D) Blink artifact. (TIF) [file pone.0210505.s001.tif]
